# RelB upregulates PD-L1 and exacerbates prostate cancer immune evasion

**DOI:** 10.1186/s13046-022-02243-2

**Published:** 2022-02-17

**Authors:** Yanyan Zhang, Shuyi Zhu, Yuanyuan Du, Fan Xu, Wenbo Sun, Zhi Xu, Xiumei Wang, Peipei Qian, Qin Zhang, Jifeng Feng, Yong Xu

**Affiliations:** 1 Laboratory of Cancer Biology, Jiangsu Cancer Hospital & Jiangsu Institute of Cancer Research & the Affiliated Cancer Hospital of Nanjing Medical University, Nanjing, 210009 China; 2 Jiangsu Key Lab of Cancer Biomarkers, Prevention and Treatment, Nanjing Medical University, Nanjing, 211166 China; 3 Department of Medical Oncology, the Affiliated Cancer Hospital of Nanjing Medical University, Jiangsu Cancer Hospital & Jiangsu Institute of Cancer Research, Nanjing, 210009 China; 4 Department of General Surgery, the First Affiliated Hospital with Nanjing Medical University, Nanjing, 210029 China; 5 Department of Surgery, Jiangsu Cancer Hospital & Jiangsu Institute of Cancer Research & the Affiliated Cancer Hospital of Nanjing Medical University, Nanjing, 210009 China

## Abstract

**Background:**

The interaction between programmed death receptor (PD-1) and its ligand (PD-L1) is essential for suppressing activated T-lymphocytes. However, the precise mechanisms underlying PD-L1 overexpression in tumours have yet to be fully elucidated. Here, we describe that RelB participates in the immune evasion of prostate cancer (PCa) via *cis/trans* transcriptional upregulation of PD-L1.

**Methods:**

Based on transcriptome results, RelB was manipulated in multiple human and murine PCa cell lines. Activated CD4^+^ and CD8^+^ T cells were cocultured with PCa cells with different levels of RelB to examine the effect of tumourous RelB on T cell immunity. Male mice were injected with murine PCa cells to validate the effect of RelB on the PD-1/PD-L1-mediated immune checkpoint using both tumour growth and metastatic experimental models.

**Results:**

PD-L1 is uniquely expressed at a high level in PCa with high constitutive RelB and correlates with the patients’ Gleason scores. Indeed, a high level of PD-L1 is associated with RelB nuclear translocation in AR-negative aggressive PCa cells. Conversely, the silencing of RelB in advanced PCa cells resulted in reduced PD-L1 expression and enhanced susceptibility of PCa cells to the T cell immune response in vitro and in vivo. Mechanistically, a proximal NF-κB enhancer element was identified in the core promoter region of the human *CD274* gene, which is responsible for RelB-mediated PD-L1 transcriptional activation. This finding provides an informative insight into immune checkpoint blockade by administering RelB within the tumour microenvironment.

**Conclusion:**

This study deciphers the molecular mechanism by which tumourous RelB contributes to immune evasion by inhibiting T cell immunity via the amplification of the PD-L1/PD-1-mediated immune checkpoint**.**

**Supplementary Information:**

The online version contains supplementary material available at 10.1186/s13046-022-02243-2.

## Background

Prostate cancer (PCa) is the most common malignant tumour and the leading cause of cancer death in men, with an estimated 1,276,000 new cases and 359,000 deaths worldwide in 2018 [1]. Owing to improved early diagnosis and advanced therapeutic strategies, the mortalities of PCa have been appreciably decreased. Unfortunately, some PCa patients eventually develop more aggressive malignant forms resistant to traditional radiotherapy and chemotherapy, leading to poor prognosis [2]. Recently, the mutation rate identified in homologous recombination DNA repair genes showed as high as 20–25% in metastatic castration-resistant prostate cancer (mCRPC) and defects in the repair genes can sensitize PCa cells to Poly(ADP-ribose) polymerase inhibitors [3]. Similar results were also found in bladder cancer [4, 5]. Notably, emerging evidence has demonstrated that enhancing the immune response is an effective therapeutic option for improving the survival of cancer patients [6]. Indeed, tumour-induced immunosuppression plays a pivotal role in tumour evasion from host immune surveillance, which provides a complementary approach to the treatment of tumours using immune checkpoint inhibitors [7]. Accordingly, multiple studies have focused on blocking immune checkpoints, such as targeting CTLA-4 and the PD-1/PD-L1 axis, which has been shown to restore or strengthen T cell antitumour immunity [8, 9]. In particular, PD-1/PD-L1 is widely recognized as the best immune checkpoint target based on its therapeutic validation in prospective clinical outcomes, including subsets of solid tumours [10].

PD-1, an important T cell co-inhibitory receptor, is expressed on the surface of antigen-stimulated T cells and plays a fundamental role in suppressing adaptive immune responses and promoting self-tolerance against T cell inflammatory activity. The activation of PD-1 prevents autoimmune diseases and impedes tumour suppression due to immunocompromise [11]. PD-1 has two known ligands, PD-L1 and PD-L2, of which PD-L1 is the dominant inhibitory ligand associated with immunotherapeutic responses within the tumour microenvironment [12]. As a critical checkpoint molecule in the regulation of the immune response, PD-1 is expressed in dendritic cells, T/B cells, tumour-associated macrophages, and myeloid-derived suppressor cells [13]. On the other hand, PD-L1 is highly expressed in a variety of cancer cells, which leads to tumour immune evasion as they interact with PD-1 [14]. Since the PD-L1/PD-1 interaction is essential for inducing T cell apoptosis and the immune checkpoint response, targeting the PD-1/PD-L1 axis is thought to be a feasible approach for treating aggressive tumours [15].

Inflammatory signalling regulates the transcription of PD-L1. In particular, IFN-γ, a proinflammatory cytokine, is recognized as the most prominent inducer of PD-L1 [16]. Additionally, several other cytokines can also upregulate PD-L1, including TNF-α, IL-1β, IL-4, IL-10, and IL-17 [17]. Multiple transcription factors involved in the JAK/STAT, RAS/MAPK, and PTEN-PI3K/AKT pathways participate in the regulation of PD-L1, such as STAT1, STAT3, IRF1, IRF3, HIF-1α, MYC, JUN, BRD4, and NF-κB [18]. Since NF-κB plays a substantial role in inflammatory and immune responses [19], NF-κB is considered to influence PD-L1 expression. RelA (p65) has been reported to upregulate PD-L1 expression in lung cancer cells in response to TNF-α stimulation [20].

NF-κB plays an essential role in cancer progression and therapeutic resistance [21]. In general, NF-κB activation is mediated by two major pathways, i. e., the canonical pathway and the noncanonical pathway. The canonical pathway quickly responds to many exogenous stimulants and leads to the nuclear translocation of the p50:RelA dimer followed by IκB phosphorylation and degradation [22]. The noncanonical pathway is gradually but persistently activated by processing p100:RelB into the p52:RelB dimer [23]. In addition to the canonical pathway that is widely recognized to be critical for regulating the inflammatory response, the noncanonical pathway is thought to be a key regulator of the immune response [24]. Nevertheless, in contrast to the well-studied canonical pathway in cancer development, the role of the noncanonical pathway in cancer remains to be fully elucidated.

RelB was initially identified in B-cells and the RelB-activated noncanonical NF-κB pathway has been shown to respond to antigen presentation by DCs, which is associated with inflammation and excessive immune cell infiltration [25–27]. Furthermore, RelB has been implicated in cancer progression, particularly in sex hormone-related cancers, including PCa, breast cancer and endometrial cancer [28–30]. We have recently shown that a high constitutive level of RelB is correlated to PCa radioresistance [31]. The present study further demonstrates a *cis/trans* transcriptional regulatory mechanism by which RelB upregulates PD-L1 in PCa cells. Accordingly, the repression of tumour-derived RelB can promote the T cell immune response by downregulating PD-L1.

## Methods

### PCa patients

The Nanjing Medical University Affiliated Cancer Hospital (Nanjing, China) collected fresh tumour tissues from newly diagnosed PCa patients before chemotherapy or radiotherapy. The Ethics Committee of Nanjing Medical University approved the study protocol with written informed consent forms obtained from patients enrolled in this study. From a Gleason score of 5 to 9, each group containing 8–10 cases was selected. Total 46 cases of PCa tumour tissue v.s 10 cases normal prostate tissue were analysed to examine the correlation between RelB and PD-L1 expression using immunohistochemistry (IHC) with monoclonal antibodies against human RelB and PD-L1 (Cell Signalling Tech., USA). Briefly, the tissues were fixed in paraffin-embedded slides and then dewaxed with xylene. After washing with ethanol, the tissue slides were further rehydrated by rinsing with dH_2_O. For IHC, the tissue slides were soaked in 5% BSA buffer for 1 h and then incubated with 400x diluted primary antibodies within 5% BSA buffer at 4 °C overnight. After washing with PBS, the tissue slides were incubated with a biotinylated secondary antibody at room temperature for 30 min. After washing with BPS, a DAB Substrate Kit (Cell Signalling Tech., USA) was used to observe immunostaining images under a microscope. The intensity of IHC staining was scored as negative (score 0), weak (1), medium (2), and strong (3). Total cell positivity was scored as the percentage of positive cells, including no positive cells (0), < 25% (1), 25–50% (2), 50–75% (3), and > 75% (4). The “H-score” was calculated using ∑pi (i + 1) for all slides, in which *pi* represented the percentage of positive and *i* represented the staining intensity.

### Mice

Animal experiments were performed according to the Institutional Animal Care and Use approved by the Research Committee of Nanjing Medical University (No. IACUC-1901031). For the mouse xenograft PCa tumour growth experiment, five-week-old male C57BL/6 mice (Beijing Vital River Laboratory Animal Technology Co., Ltd., China) were randomly divided into several groups. The mice were subcutaneously injected with 2.5 × 10^5^ RM-1 cells with different levels of RelB and PD-L1 into the left flank. The formed tumours were measured using digital callipers every other day and tumour volume was calculated using a standard formula (V = 0.52 × AB^2^, where A and B represent the diagonal tumour lengths) at 7, 14 and 21 days after the cell injection. CD4^+^ and CD8^+^ T cells were isolated from mouse blood samples and analysed by flow cytometry. The mice were finally sacrificed and tumour tissues were excised for immunoblotting and IHC. For the xenograft PCa tumour metastasis experiment, 5 × 10^4^ RM-1 cells were injected into mice through the tail vein. The mice were euthanized four weeks later to remove lung tumour tissues and analysed by IHC and immunoblots. Additionally, CD4^+^ and CD8^+^ T cells isolated from mouse spleen tissues were analysed by flow cytometry.

### T cell preparation

Whole blood samples were obtained from healthy donors according to the institutional guidelines with informed consent. Peripheral blood mononuclear cells (PBMCs) were isolated by density centrifugation using Ficoll gradient separation. The cells were washed three times with PBS and suspended in RPMI 1640 media (Gibco, USA). In addition, mouse T cells were isolated from peripheral blood samples and spleen tissues derived from male C57BL/6 mice. The pellets were lysed within RBC lysis buffer for 10 min to remove red blood cells and the tissues were ground and then filtered to remove tissue debris. The cells were cultured in a T cell preconditional RPMI 1640 media supplemented with 1000x mercaptoethanol (Gibco), 100x penicillin-streptomycin (Gibco), 100X glutamine-MAX (Gibco) and 10% heat-inactivated FBS (Gibco). Single cells were collected by centrifugation and T cells were stimulated in media containing 2 μg/ml anti-CD3, 3 μg/ml anti-CD28 and 200 U/ml IL-2 (Biolegend, Inc., USA) for 24 h prior to coculture with PCa cells.

The cultured T cells were analysed by flow cytometry before and after coculture with PCa cells. APC-conjugated anti-human CD4/mouse antibody (Biolegend) and PE-conjugated anti-human CD8/mouse antibody (BioLegend) were used to stain CD4 or CD8 cells. The labelled cells were analysed by a BD FACSCalibur flow cytometer (BD Sciences, USA). In addition, to analyse T cell proliferation, the T cells were labelled with 5 μM CFSE (Biolegend) for 20 min at 37 °C in darkness. Cell staining was blocked by adding cell culture media containing 10% FBS. Flow cytometry data were further analysed with FlowJo software and Modfit software.

### RelB manipulation in PCa cells

Human PCa cell lines (PC-3 and DU-145) and mouse PCa cell lines (RM-1) were purchased from the American Type Culture Collection (ATCC, USA). The cell lines were cultured in the recommended media containing 10% FBS and 1% penicillin/streptomycin. The cells were treated with IFN-γ (Novus, USA) to induce PD-L1 expression. RelB was silenced in PC-3 and DU-145 cells by transfection of a plasmid-carrying shRNA duplex targeting RelB (RiboBio Co., Ltd., China) and stable cell clones were selected using G418 (Invitrogen, USA**)**. Additionally, to restore RelB activity in RelB-defective PC-3 cells, a RelB cDNA driven by the CMV promoter in pCMV-Script vector (Stratagene, USA) was transiently transfected into RelB-silenced cells. Furthermore, RelB was knocked out (RelB-KO) in RM-1 cells using a CRISPR/Cas9-based gene-editing system. Briefly, RM-1 cells were transfected with a Cas9-single guide RNA (sgRNA) expression plasmid targeting RelB (sgRNA, 5′-GACGAATACATTAAGGAGAA-3′), followed by puromycin selection. In addition, PD-L1 cDNA was cloned into the pcDNA plasmid and then transfected into RelB-KO RM-1 cells, followed by hygromycin B (Invitrogen) selection to generate a stable cell line.

### Immune cytotoxicity

The effect of activated T cells on the survival of PCa cells was determined using cell counting and clonogenic assays, respectively. After coculture with activated T cells at a 1:10 ratio for 3 days, PCa cells were seeded into 96-well plates at a density of 10^3^ cells/well and then cultured for 2 days. The cells were treated with CCK-8 reagent (Dojindo Mol. Tech., Japan), and cell viability was measured as the optical density at 450 nm. For the clonogenic assay, PCa cells were treated with an anti-PD-L1 mAb (Abcam, USA) prior to coculture with activated T cells. Thereafter, 100–200 PCa cells were plated in 6-well plates and continuously cultured to allow colony formation. After washing with 1x PBS twice, the colonies were stained with 1% crystal violet dye for 30 min to form visible cell clones. The cell survival fractions were calculated based on the number of colonies divided by the number of cells efficiently plated.

### RNA-seq

The RNA sequencing libraries were constructed by Vazyme Biotech Co., Ltd. (Nanjing, China) using a VAHTS mRNA-seq v2 Library Prep Kit for Illumina. The cDNA libraries were sequenced on an Illumina HiSeq X Ten platform with a 150 bp paired-end module. The raw reads were filtered by removing reads containing adapter, poly-N and low-quality read for subsequent analysis. Cuffdiff (v1.3.0) was used to calculate FPKMs for coding genes in each sample. Genes with corrected *p*-value less than 0.05 and the absolute value of log2 (fold change) greater than or equal to 1 were considered as significantly differentially expressed. Custom scripts in R software were used for clustering and heatmap analysis (https://www.r-project.org). In addition, the altered mRNA expression profile in shRelB cells vs. shCtrl cells was analysed using KEGG pathway enrichment (https://david.ncifcrf.gov).

### Immunofluorescence

PCa cells were seeded in confocal dishes at a density of 2 × 10^3^, washed with cold 1x PBS and fixed with 4% paraformaldehyde in PBS for 15 min. Subsequently, the cells were permeabilized with 1x PBS containing 0.5% Triton X-100 for 20 min and then blocked for 30 min. The cells were incubated with primary antibodies against RelB and PD-L1 at 4 °C overnight and then probed with Alexa Fluor® 647 conjugated (Cell Signalling Tech., USA) or Alexa Fluor® 488 conjugated (Abcam, UK) secondary antibodies for 1 h at room temperature. After counterstaining with DAPI (Invitrogen) for 5 min, fluorescence was visualized and captured using a TCS SP5 MP confocal microscope (Leica Microsystems, Inc., USA).

### NF-κB binding activity

Nuclear proteins were extracted from PCa cells using a nuclear and cytoplasmic protein extraction kit (Beyotime Biotech.) according to the manufacture’s instruction. The nuclear extracts (50 μg) were subjected to an NF-κB binding activity kit containing the consensus sequence of the NF-κB element as a standard probe (Abcam, USA) to measure the NF-κB binding activity based on the manufacturer’s protocol.

### Luciferase reporter assay

A 2000-bp 5′-flanking region of the human *CD274* gene containing a core promoter containing putative multiple NF-κB elements was cloned into the pGL3 vector (Promega, USA) to drive the *luciferase* reporter gene. Subsequently, a functional NF-κB binding site was mutated using a site-directed mutagenesis system (Thermo Fisher, USA). The luciferase reporter constructs were cotransfected with β-gal into PC-3 cells using Lipofectamine (Invitrogen). After 48 h, the luciferase activity was quantified by a luciferase assay system (Promega, USA) using a luminometer (Berthold Tech., Germany). The β-gal activity was quantified using microplate readers (BioTek, USA). NF-κB-mediated transcriptional enhancement was estimated by the β-gal-normalized luciferase response.

### ChIP

RelB binding to the NF-κB elements in the human *CD274* gene was examined by chromatin immunoprecipitation (ChIP) using a SimpleChIP Enzymatic Chromatin IP Kit (Cell Signalling Tech.). A RelB antibody (Santa Cruz Biotech., USA) was applied to pull down chromatin from nuclear extracts isolated from PCa cells. Chromatin without antibody pulldown served as the input control, and IgG (Santa Cruz Biotech.) served as a negative antibody control. The pulled-down fragments were quantified by PCR using relative primers (Table S1). The amounts of the pulled-down fragments were assessed by normalization to the input control.

### EMSA

Electrophoretic mobility shift assay (EMSA) was further performed to confirm the specific RelB binding to the human *CD274* promoter. A double-stranded DNA fragment containing the native or mutated NF-κB binding site were synthesized and annealed. The 3′- terminus of the upper strand was labelled using a probe biotin-labelling kit (Beyotime Biotech., China). EMSA was conducted by incubating the probes with PC-3 cell-derived nuclear proteins within a chemiluminescent EMSA kit (Beyotime) according to the manufacturer’s instructions. The unlabelled wild-type and mutated probes were used as competitors to quantify the specific binding activity. A RelB antibody was used to eliminate the binding activity. The EMSA image was visualized using a BioRad *Gel*Doc XR+ system. The sequences of the wild-type and mutant probes are listed in Table S1.

### RT-qPCR

The mRNA levels of RelB and PD-L1 were quantified by **r**everse **t**ranscription-quantitative PCR (RT-qPCR). Total RNA was extracted from PCa cells using the TRIzol reagent and cDNA was reversely transcribed from mRNA using a PrimeScript™ RT reagent kit (Takara Inc., Japan) according to the manufacturer’s instructions. cDNA was quantified by qPCR with SYBR Premix Ex Taq II (Takara Inc.) using a LightCycle System (Roche, USA). The mRNA level of PD-L1 was estimated by normalizing to GAPDH mRNA. Sequences of the specific PCR primers for PD-L1 and GAPDH are listed in Table S1.

### Immunoblots

PCa tumour tissues and cells were lysed in RIPA lysis buffer containing 1 mM PMSF protein inhibitor (Santa Cruz Biotech.). Cellular and nuclear extracts (50–100 μg) were separated on 10% SDS-polyacrylamide gels and then transferred to PVDF membranes. The membranes were subsequently incubated overnight at 4 °C with the primary antibodies against RelA, RelB, PCNA, and GAPDH (Cell Signalling Tech.), and anti-human PD-L1, anti-mouse PD-L1 (Abcam). The membranes were washed three times with TBST buffer and incubated at room temperature for 2 h with HRP-conjugated secondary antibody (Santa Cruz Biotech. USA). The immunoblots were visualized using an enhanced chemiluminescence detection system (Bio-Rad, USA). The intensities of the blots were quantified using Quantity One software and protein expression was normalized to loading controls such as β-actin and GAPDH.

### Bioinformatics

Oncomine™ (https://www.oncomine.org) and TCGA (https://www.cancer.gov/about-nci/organization/ccg/research/structural-genomics/tcga) datasets were examined to assess the association of RelA, RelB or PD-L1 expression profiles with cancer progression and the correlation of RelA/RelB to PD-L1 was analysed to estimate the possibility that NF-κB regulates PD-L1 expression in tumours.

### Statistics

The results are presented as the mean ± standard deviation (SD) from at least three replicates. Significant differences between the experimental groups were analysed by unpaired Student’s t-test. One-way analysis of variance (ANOVA) followed by Dunnett’s or Bonferroni’s multiple comparison test was performed using Prism (GraphPad, San Diego, USA). Statistical significance was accepted at *P* < 0.05.

## Results

### The constitutive levels PD-L1 and RelB are correlated with the aggressiveness of PCa

RelB is associated with PCa and breast cancer progression [29, 30]. In consideration of the putative role of RelB in the regulation of cancer immune escape, we examined the Oncomine™ and TCGA datasets to enrich the potential relationship between PD-L1 and RelB signatures in cancer progression. PD-L1 is uniquely expressed at high levels in multiple cancer tissues compared to their corresponding normal tissues (Fig. S1a). In addition, the correlation of PD-L1 with RelA and RelB in PCa was assessed. The results indicated that PD-L1 is more highly associated with RelB than RelA in PCa tumour tissues, but no clear correlation was found in peritumoral tissues (Fig. S1b). Interestingly, similar results were observed in breast cancer, thus indicating that the noncanonical NF-κB pathway is critical for developing sex hormone-related cancers (Fig. S1c). In contrast, the correlation of PD-L1 with RelA is higher than that of RelB in lung cancer. There was no apparent relationship of PD-L1 with RelA or RelB in liver cancer (Fig. S1d-e).

**Fig. 1:**
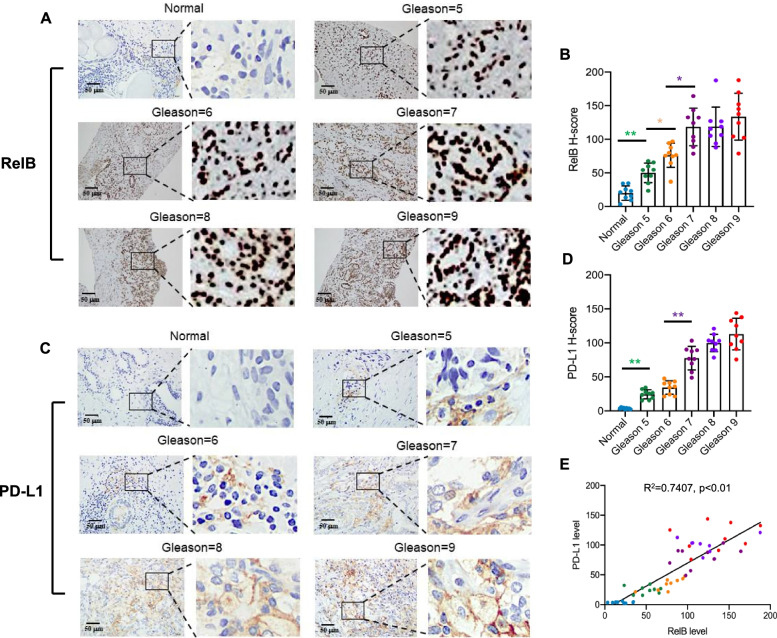
PD-L1 is correlated with RelB in PCa progression. **a-b** Normal prostate and PCa tissues were examined to determine the association between RelB expression and patients’ Gleason scores by IHC. **c-d** Correspondingly, the association between PD-L1 expression and patients’ Gleason scores was analysed. **e** Linear regression analysis determined the correlation of RelB and PD-L1 in PCa progression. The H-scores of IHC were plotted and *(*p* < 0.05) and **(*p* < 0.01) show significance between the two groups as indicated

To verify whether the correlation of PD-L1 and RelB is also associated with the aggressiveness of PCa, tumour and normal prostate tissues were examined by IHC with specific antibodies against RelB and PD-L1. As expected, PD-L1 and nuclear RelB were consistently elevated, which corresponded to an increase in patients’ Gleason scores (Fig. 1a-d). The correlation between PD-L1 and RelB was associated with the pathological grades of tumour tissue samples (Fig. 1e). Thus, the results are consistent with the results from the Oncomine™ database and suggest that RelB may participate in the regulation of the *CD274* gene expression during PCa progression.

### RelB upregulates PD-L1 expression in PCa cells

**Fig. 2:**
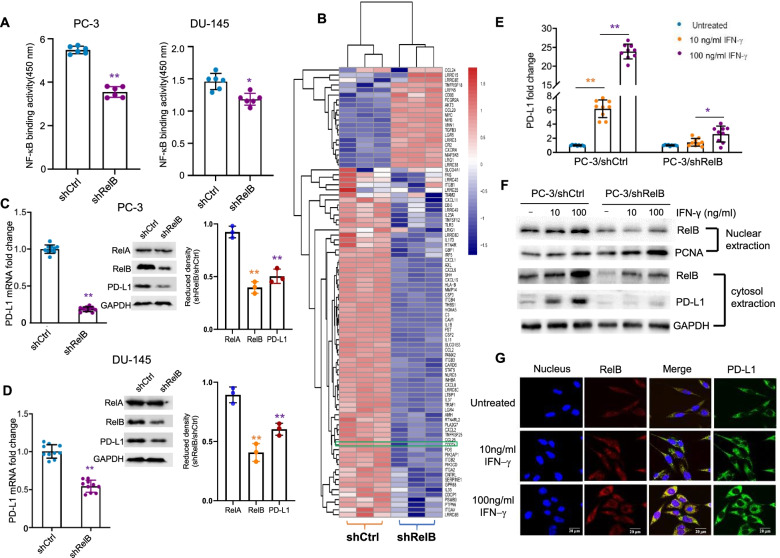
RelB regulates PD-L1 in PCa cells. **a** RelB was silenced in PC-3 and DU-145 cells using a lentiviral shRNA specifically targeting endogenic RelB. The NF-κB binding activities in RelB-silenced cells (shRelB) vs. scramble sequence (shCtrl) were measured using an ELISA kit with a standard probe containing the consensus NF-κB element. **b** mRNA expression profiles of RelB-silenced cells vs. control cells were examined by RNA sequencing. The relative levels of inflammatory and immune responsive transcripts were analysed, as indicated by the heatmap. The signature of *CD274* gene expression was indicated in a green box. **c-d** The levels of RelA, RelB and PD-L1 mRNA and proteins were quantified using RT-qPCR and immunoblots by normalizing to GAPDH. **e** PC-3 cells were treated with different doses of IFN-γ as indicated. The stimulated PD-L1 mRNA expression was quantified by RT-qPCR with GAPDH normalization. **f** After IFN-γ treatment, the levels of nuclear RelB and relative cytosolic PD-L1 in PC-3 cells were measured by immunoblots. PCNA served as an internal control for the nuclear fraction, and GAPDH served as a loading control for the cytosolic fraction. **g** Accordingly, RelB nuclear translocation and relative PD-L1 in the cytosol were confirmed using a confocal microscope. *(*p* < 0.05) and **(*p* < 0.01) show significance between the two groups as indicated

Although the activation of the RelA-based canonical NF-κB pathway is involved in chemo- and radiotherapy-mediated PD-L1 induction in cancer cells [32, 33], there is no evidence showing that the RelB-based noncanonical pathway contributes to PD-L1 expression in cancers. To elucidate the effect of RelB on PCa immune evasion, RelB was silenced in two aggressive AR-negative PCa cell lines (PC-3 and DU-145) using a lentiviral shRelB expressing construct. The reduction of NF-κB binding activity in RelB-silenced cells was further confirmed. However, since other NF-κB members can also bind to the probe with the consensus NF-κB sequence, the effect of RelB silencing on total NF-κB binding activity was diluted, especially in DU-145 cells (Fig. 2a). RNA-Seq was applied to analyse mRNA expression profiles in the RelB-silenced PC-3 cells. The expression profiles of transcripts relevant to cytokine/chemokine production, inflammatory signalling pathways, and immune responses were selected, as illustrated in Fig. 2b. Compared to the scramble control cells, most mRNA expression levels were reduced in response to the silencing of RelB. KEGG-enriched signalling pathway analysis showed that the silencing of RelB led to reductions in advanced PCa-associated cytokines/chemokines (Fig. S2a). Inflammation recognition-associated TLRs, NLRs and LTM were also decreased in RelB-silenced cells (Fig. S2b). Additionally, the silencing of RelB in PCa cells led to the inhibition of receptors associated with immune response (Fig. S2c). Furthermore, the mRNA expression profiles related to the immune response in the RelB-silenced cells are listed in Table S2. Remarkably, the mRNA level of the *CD274* gene was decreased in the RelB-silenced PC-3 cells.

To verify that RelB is able to regulate PD-L1 in PCa cells, the mRNA and protein levels of PD-L1 in RelB-silenced PC-3 and DU-145 cells were quantified. RelB was efficiently knocked down in the two cell lines, but no change in RelA expression was observed. Consistent with the decline of RelB, PD-L1 mRNA and protein levels were also reduced (Fig. 2c-d). In addition, IFN-γ remarkably induced PD-L1 expression in PC-3 cells, but the induction was alleviated when RelB was silenced (Fig. 2e). Consistent with the decreased level of nuclear RelB, the induction of PD-L1 protein was impeded (Fig. 2f). The correlation of nuclear RelB and cytosolic PD-L1 was further confirmed by confocal microscopy (Fig. 2g). These results suggested that IFN-γ induces PD-L1 expression partially via RelB-mediated transcriptional activation.

### A proximal NF-κB element was identified to be responsive to RelB-mediated the *CD274* gene transcriptional regulation

**Fig. 3:**
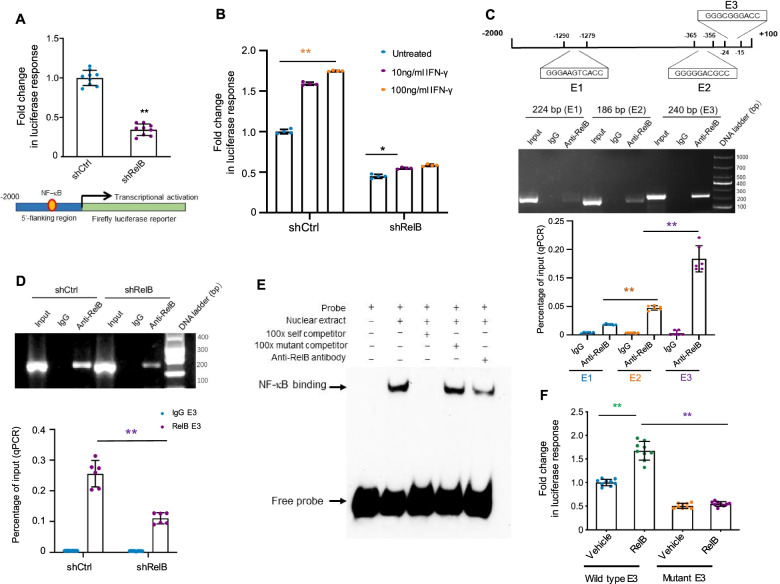
RelB regulates PD-L1 in PC-3 cells. **a** A 2000-bp flanking region of the human *CD274* gene containing putative NF-κB elements and a core promoter was cloned to drive the *luciferase* reporter gene, as indicated in the lower panel. The reporter construct was transfected into PC-3 cells with different levels of RelB and the transcriptional activity was estimated by β-gal-normalized reporter responses. **b** After cell transfection, the cells were treated with IFN-γ as indicated. Τhe IFN-γ-mediated transcriptional induction was analysed by the reporter assay. **c** Chromatin was precipitated using a RelB antibody, and DNA fragments containing three putative RelB elements (E1, E2, E3) were quantified by regular PCR (upper panel) and quantitative PCR (lower panel). Chromatins without the IP procedure were amplified as input controls. IgG was used as a negative antibody control. **d** Chromatins derived from RelB-silenced cells were pulled down and the reduction in the E3 fragment was quantified. **e** A 24-bp double-stranded DNA fragment containing the E3 element was synthesized for the preparation of an EMSA probe with biotin-labelling. Nuclear extract was incubated with the probe and the specific NF-κB binding was determined by EMSA with a self competitor. Additionally, the NF-κB element in the E3 fragment was mutated as a mutant competitor (shown in Fig. S3a). Furthermore, the RelB antibody was used to specifically reduce the E3 binding activity. **f** The mutated E3 binding site was further cloned and its effect on RelB-mediated transcriptional activation was assessed by the reporter assay. *(*p* < 0.05) and **(*p* < 0.01) show significance between the two groups as indicated

Although several lines of study have demonstrated that NF-κB signalling regulates PD-L1 expression, there is a lack of experimental evidence that NF-κB directly regulates PD-L1 expression in a *cis/trans* transcriptional regulatory manner. To elucidate how RelB regulates *CD274* gene expression, a 2000-bp 5′-flanking fragment region containing a core promoter was cloned to drive *luciferase* reporter gene expression. Luciferase activity was reduced in RelB-silenced PC-3 cells (Fig. 3a). Additionally, the IFN-γ-induced reporter response declined when RelB was silenced (Fig. 3b). Three putative NF-κB binding sites (E1, E2, E3) located in the 5′-finking region were identified by analysing the Jaspar transcription factor database (http://jaspar.genereg.net). Accordingly, a ChIP assay was performed to verify each binding site using a RelB antibody. As shown in Fig. 3c, the proximal site (E3) appeared to be more susceptible to ChIP than E2 and distant E1 sites. The amount of E3 pull-down element was consistently reduced when chromatin extracted from RelB-silenced cells was used (Fig. 3d). Moreover, RelB binding to the E3 site was validated by EMSA. The nuclear extract was capable of shifting the probe containing the E3 site but not the mutant E3 site. An unlabelled probe was able to compete with E3 binding, but an unlabelled mutant probe was found to have no such competition. In addition, the RelB antibody was able to reduce the E3 binding activity (Fig. 3e). Furthermore, cell transfection with the mutated E3 site resulted in a reduction in the RelB-activated reporter response (Fig. S3a and Fig. 3f). Altogether, these results suggest that RelB transcriptionally regulates the human *CD274* gene expression via a proximal NF-κB element located in the core promoter region, which is conserved in humans, mice and rabbits (Fig. S4a-b).

### Tumor-derived RelB contributes to inactivation of CD4^+^ and CD8^+^ T cells

**Fig. 4:**
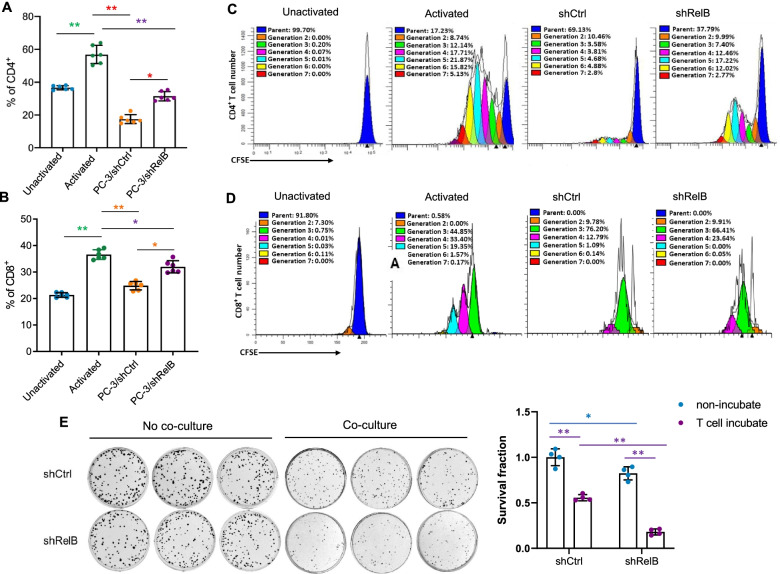
Silencing RelB in PC-3 cells enhances the immunities of CD4^+^ and CD8^+^ T cells. **a-b** PC-3 cells were cocultured with activated T cells derived from human PBMCs. CD4^+^ and CD8^+^ T cells were quantified by flow cytometry using the relevant antibodies. **c-d** Subsequently, the proliferation of CD4^+^ and CD8^+^ T cells was analysed by flow cytometry. The percentage of each generation (cell division) was plotted. **e** After coculture with activated T cells, the survival rate of PC-3 cells was measured using a clonogenic assay. *(*p* < 0.05) and **(*p* < 0.01) show significance between the two groups as indicated

Cancer immunotherapy aims to promote the cytotoxic effect of T lymphocytes within the tumour microenvironment. The signalling process is mainly relayed from CD4^+^ and CD8^+^ T cells by specific dendritic cells to optimize the immune response of T lymphocytes [34, 35]. Thus, it is important to define the immune responsiveness of T cells to RelB-depleted PC-3 cells. To this end, we collected blood samples and isolated primary T cells from healthy donors who participated in this study. T cells were activated by pretreatment with anti-CD3, anti-CD28 and IL-2, and then cocultured with RelB-silenced PC-3 cells. The percentages of CD4^+^ and CD8^+^ T cells and their proliferation were quantified by flow cytometry with relative specific antibodies and CFSE dye. The results showed that stimulation by CD3 and CD28 efficiently increased the numbers of T cells. Interestingly, coculture with PC-3 cells significantly reduced the activated CD4^+^ and CD8^+^ T cells, but its effect was alleviated when RelB was depleted in PC-3 cells (Fig. 4a-b). In addition, the proliferation of CD4^+^ and CD8^+^ T cells was consistently increased by stimulation but further precluded after coculturing with PC-3 cells. However, the inhibitory effect of PC-3 cells on T cell activation was favourably diminished by abrogating RelB (Fig. 4c-d).

Furthermore, to test whether tumourous RelB promotes T cell immune compromise by increasing PD-L1**,** the survival of PC-3 cells was quantified by clonogenic assay after coculture. The activated T cells efficiently eliminated the PC-3 cell colony number compared to the no coculture control. Intriguingly, the T cell immune response was further enhanced when RelB was silenced in PC-3 cells, which suggests that the high level of RelB in PC-3 cells contributes to immune evasion (Fig. 4e). Moreover, RelB was restored in lentivirus-mediated RelB-silenced PC-3 cells by transiently transfecting a human RelB cDNA expression construct into the cells (Fig. S5a). After coculture with activated T cells, the cancer cell survival rate fully recovered as RelB expression was restored (Fig. S5b). Accordingly, the numbers of active CD4^+^ and CD8^+^ T cells were apparently reduced by increasing RelB in the cancer cells (Fig. S5c-e).

### RelB deprivation enhances immune checkpoint blockade by an anti-PD-L1 inhibitor

**Fig. 5:**
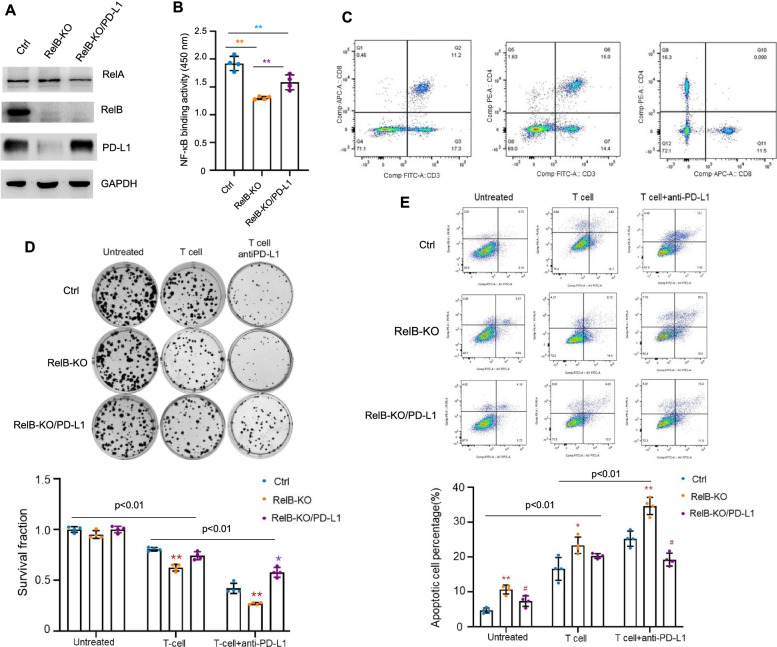
RelB deprivation in mouse PCa RM-1 cells restores mouse T cell function. **a** RelB was knocked out in RM-1 cells using the CRISPR/Cas9-based gene-editing system and further PD-L1 was enforcedly expressed in RelB-deprived cells to restore the immunocompromise. The cellular levels of RelA, RelB and PD-L1 proteins in these cell lines were measured by immunoblots. **b** Subsequently, the NF-κB binding activities in the established cell lines were quantified. **(*p* < 0.01) shows significance between the two groups as indicated. **c** T cells derived from mouse spleen tissues were activated and CD4^+^ and CD8^+^ T cells were quantified by flow cytometry. **d** RM-1 cells were treated with an anti-PD-L1 mAb prior to coculture with activated T cells, and the RM-1 cell survival was measured using a clonogenic assay. The significance between the two groups as indicated on the tap. Within the same groups, *(*p* < 0.05), **(*p* < 0.01) shows significance in RelB-KO and RelB-KO/PD-L1 cells compared to control cells. **e** Correspondingly, the apoptosis of RM-1 cells was further analysed using flow cytometry. The significance between the two groups as indicated on the tap. Within the same groups, *(*p* < 0.05), **(*p* < 0.01) show significance in RelB-KO cells vs. the control cells, and ^#^(*p* < 0.05) shows significance in RelB-KO/PD-L1 cells compared to control and RelB-KO cells

Meanwhile, a CRISPR/Cas9 gene-editing system was applied to knock out RelB in murine PCa RM-1 cells to verify that RelB contributes to immune evasion in vivo. Additionally, mouse PD-L1 was ectopically expressed in RelB-KO cells to restore immune suppression (Fig. S3b and Fig. 5a). NF-κB binding activity was measured in gene manipulated RM-1 cells (Fig. 5b). T cells were prepared from mouse spleens and then activated by CD3 and CD28 stimulation before coculturing with RM-1 cells (Fig. 5c). Consistent with the above results from PC-3 cells, RelB-KO cells were more susceptible to T cells than were the control cells, but T-cell activation was further eliminated by enforced expression of PD-L1 in RelB-KO cells (Fig. S6a). Similarly, coculture with RelB-KO cells led to recovered CD4^+^ and CD8^+^ T cells compared to coculture with RM-1 cells. In turn, the RelB-KO effect was further attenuated by expressing PD-L1 (Fig. S6b-c). In addition, the proliferation of CD4^+^ and CD8^+^ T cells further confirmed that T cell growth was virtually regulated by administrating RelB and PD-L1 in RM-1 cells (Fig. S6d-e).

Moreover, after coculture with activated T cells, RM-1 cells were treated with an anti-PD-L1 mAb. The results showed that the mAb dramatically enhanced the immune response of the T cells. Notably, RelB deprivation further enhanced mAb therapeutic efficiency. Nevertheless, the enforced expression of PD-L1 in the RelB-KO cells led to partial rescue of the cells (Fig. 5d). Correspondingly, the apoptotic cell rate was increased in RelB-KO cells treated with T cells plus an anti-PD-L1 inhibitory mAb, but the effect was further alleviated as the PD-L1 level was elevated (Fig. 5e). These results suggest that the immune checkpoint blockade of PD-1/PD-L1 can be modulated by manipulating RelB.

### RelB contributes to PCa tumour immune evasion in mice

**Fig. 6:**
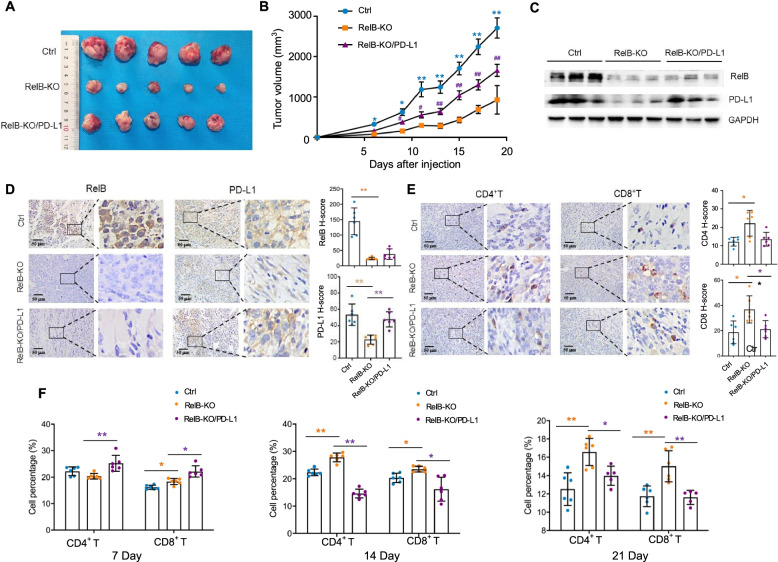
RelB deprivation reduces mouse xenograft tumour growth. **a** RM-1 (control), RM-1:RelB-KO and RM-1:RelB-KO/PD-L1 cell lines were cultured and then subcutaneously injected into male C57BL/6 mice for tumour formation. After tumours reached the maximal volume, the mice were sacrificed and the excised tumour tissues were photographed. **b** Tumour volume was measured every other day, and the tumour growth rate was determined as plotted in each group. *(*p* < 0.05), **(*p* < 0.01) show the significance in RelB-KO cells and RelB-KO/PD-L1 compared to the control cells; and ^#^(*p* < 0.05), ^##^(*p* < 0.01) indicate the significances in RelB-KO/PD-L1 cell vs. RelB-KO cells. **c** The expression levels of RelB and PD-L1 in the tumour tissues were measured by immunoblots. **d-e** The levels of RelB, PD-L1, CD4 and CD8 proteins in the tumour tissues were quantified by IHC and the relative H-scores were plotted. *(*p* < 0.05), **(*p* < 0.01) show significance between the two groups as indicated. **f** Blood samples were collected from mice on different days after cell injection as indicated to analyse the percentages of CD4^+^ and CD8^+^ T cells by flow cytometry. *(*p* < 0.05), **(*p* < 0.01) show significance between the two groups as indicated

A mouse tumour xenograft model was applied to define the role of RelB in the immune checkpoint of PD-1/PD-L1. RM-1, RM-1:RelB-KO and RM-1:RelB-KO/PD-L1 cell lines were used for tumour formation by subcutaneous injection into mice. In the control group, 3–5 days after injection, tumours were formed and then rapidly grew to reach the maximal tumour volume (3000 mm^3^) within three weeks. In contrast, tumour formation in the RelB-KO group was delayed and tumour growth was also slow. Nevertheless, the tumour growth in the RelB-KO/PD-L1 group was restored due to increased PD-L1 (Fig. 6a-b). All mice were sacrificed when the average tumour volume in the control group reached the maximum and tumour tissues were excised. Regardless of endogenous mouse PD-L1, the levels of PD-L1 in RelB-KO tumours were significantly reduced, but the levels were raised in RelB-KO/PD-L1 tumours (Fig. 6c-d). According to the manipulated PD-L1, the levels of CD4 and CD8 proteins increased via knockout of RelB, but the levels further decreased as PD-L1 was expressed (Fig. 6e). In addition, mouse serum samples were collected for T-cell activation (Fig. S7a). Compared to the control group, the numbers of T cells were increased in the RelB-KO group (Fig. S7b). After tumour formation, the amounts of CD4^+^ and CD8^+^ T cells slightly increased, but in turn, they rapidly decreased as the tumour consistently grew. Although CD4^+^ and CD8^+^ T cells were high in the RelB-KO group, the cell numbers declined as PD-L1 expression increased in tumour cells (Fig. 6f).

**Fig. 7:**
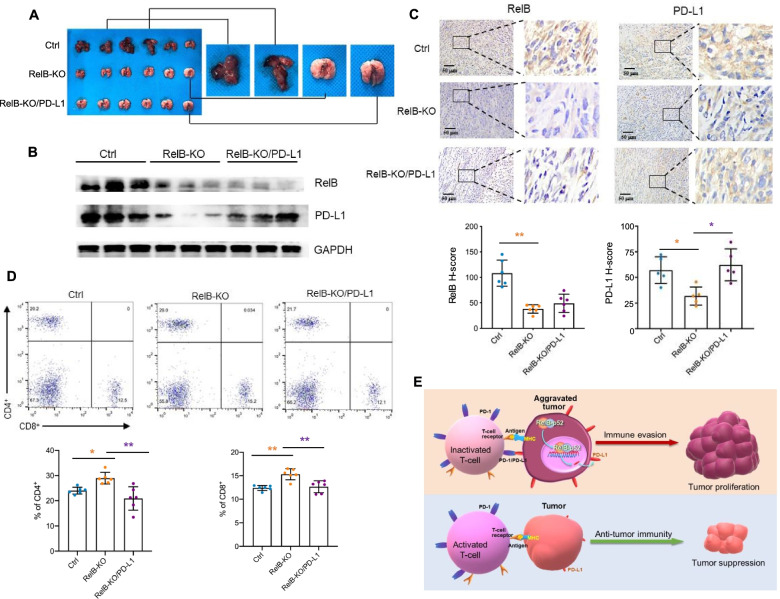
RelB deprivation decreases mouse xenograft tumour lung metastasis. **a** RM-1 and its generated cell lines were intravenously injected into male C57BL/6 mice. Lung tissues with tumours in the control group and without tumours in the RelB-KO groups were excised and examined. **b** The expression levels of RelB and PD-L1 in the excised tissues were measured by immunoblots. **c** Additionally, the expression of RelB and PD-L1 was further quantified by IHC and the relative H-scores were plotted. **d** The percentages of CD4^+^ and CD8^+^ T cells derived from mouse blood samples were analysed by flow cytometry. *(*p* < 0.05), **(*p* < 0.01) show significance between the two groups as indicated. **e** Depiction of the suggested mechanism underlying RelB-mediated immune evasion of PCa cells

Moreover, to examine whether RelB deprivation also inhibits PCa metastasis by downregulating PD-L1, the three tumour cell lines were further injected into mice through the tail vein. Three weeks after the injection, metastatic lung tumours were detected in the control group, but not in the groups injected with RelB-KO tumour cells irrespective of PD-L1 expression in the cells, thus indicating that RelB is critical for PCa metastasis (Fig. 7a). Furthermore, consistent with the reduction in RelB, the PD-L1 levels also decreased in the RelB-KO group but increased in the PD-L1-expressing group (Fig. 7b-c). Accordingly, CD4^+^ and CD8^+^ T cell numbers in serum increased in mice injected with RelB-KO RM-1 cells, but the T cell number decreased in injected mice by restoring PD-L1 in RM-1 cells (Fig. 7d). Taken together, the results from the present study delineated that the high levels of RelB in advanced PCa cells promote immune evasion by transcriptional upregulation of PD-L1, as illustrated in Fig. 7e.

## Discussion

Although the 5-year survival rates of PCa have steadily increased in the United States, the mortality of PCa has been consistently increasing globally, particularly in East Asia [2]. Moreover, AR-negative metastatic PCa displays resistance to the most common treatments and leads to poor prognosis; in particular, devastating bone metastasis appears to be a salient and daunting challenge in the control of PCa [36]. Thus, a comprehensive therapeutic strategy in combination with innovative technologies urgently needs to conquer intractable malignant PCa. In this regard, the advantages of novel cancer immunotherapy have emerged as a prospective method for treating worsened tumours, including advanced PCa [10, 37].

Mounting evidence has demonstrated that immune checkpoint inhibitors have significantly improved overall survival for subsets of patients with malignant tumours that mostly resist traditional anticancer therapies [38, 39]. For instance, the therapeutic effects of the blockade of PD-1/PDL-1 and CTLA-4 checkpoints have been adopted to treat various cancers [40, 41]. PD-L1 is uniquely expressed at high levels in cancers; therefore, therapies targeting PD-1/PD-L1 have been shown to promote remarkable antitumour immunity and acquire promising therapeutic outcomes for several malignant tumours [14, 42]. Likewise, PD-L1 is profoundly expressed in tumour tissues from mCRPC patients, which suggests that PD-L1 is associated with PCa progression [43]. Although anti-PD-1 treatment has shown therapeutic results for treating primary PCa, the combination of anti-PD-1 mAb with myeloid-derived suppressor cell (MDSC)-targeted therapy has received a robust synergistic therapeutic response in the treatment of mCRPC [44]. Nevertheless, less response to anti-PD-L1 agents in metastatic urothelial cancer and mCRPC suggested stromal TGF-β dampening the PD-1/PD-L1 blockade therapy, which indicates the existence of alternative immunosuppressive mechanisms provided from the tumour microenvironment [45, 46].

It is recognized that pro-inflammatory cytokines produced in the tumour microenvironment induce PD-L1 expression in tumour cells [47, 48]. In particular, IFN-γ has been defined as a favourable adaptive immune resistance inducer. Other oncogenic cytokines are also involved in inducing PD-L1 expression, including TGF-β, IL-6, IL-10, and IL-17 [18]. Mechanistically, the expression of PD-L1 in tumour cells can be regulated through transcriptional and posttranscriptional regulation [17]. Several cytokine/chemokine-inducible transcription factors have been shown to participate in the regulation of PD-L1, such as Myc, NF-κB, Stat3, and Jun/Ap-1 [18, 49]. Additionally, tumour-suppressive miR-34a appeared to directly downregulate PD-L1 [50], while oncogenic miR-21 seemed to indirectly upregulate PD-L1 via activation of the PI3K-Akt signalling axis by inhibiting PTEN [51]. Furthermore, a recent study demonstrated that AKT upregulates PD-L1 by phosphorylating β-catenin and promotes glioblastoma immune evasion [52].

Transcriptional regulation plays a pivotal role in PCa progression. AR-mediated transcriptional regulation plays a fundamental role in the promotion of AR-dependent PCa [53]. Nevertheless, PCa patients frequently receive androgen deprivation and AR-inhibitory therapies, and the AR response is ultimately eradicated in the developed AR-negative malignancy [54, 55]. Notably, NF-κB-mediated transcriptional regulation, in turn, to be activated in mCRPC, functionally sustains PCa progression under androgen-free conditions [56]. Although the activation of the RelA-based canonical NF-κB pathway has been involved in PCa progression and therapeutic resistance [57, 58], the role of the RelB-based noncanonical NF-κB pathway is underestimated.

RelA has been reported to upregulate PD-L1 in tumour cells in response to TNF-α and LPS stimulation or by cooperation with RB phosphorylation [20, 59, 60]. Nevertheless, there is a lack of current evidence that p50/RelA directly binds to the NF-κB element located in the *CD274* promoter. Recently, Antonangeli et al. predicted the canonical NF-κB consensus sequence in the *CD274* promoter region as 5′-GGGRNWYYCC-3′ (where R: A/G, W: A/T, Y: C/T, N: any base) [61]; however, the proposed site has yet not fully validated. The present study used the standard NF-κB consensus sequence (5′-GGGRNYYYCC-3′) to search the potential NF-κB binding site in the *CD274* promoter. Three putative sites were validated using a systemic *cis/trans* transcriptional regulatory approach. The results delineated that a proximal NF-κB enhancer element located in the core promoter region is responsive to RelB-mediated PCa immune evasion by upregulating PD-L1. Consistently, the silencing of tumourous RelB led to enhanced T cell immunity in the suppression of PCa.

Notably, the RelB-based noncanonical NF-κB pathway has been implicated in diverse biological processes, including immunogenicity and tumorigenicity [62]. RelB function is critical for normal B cell maturation and lymphoid organogenesis [63, 64]. BAFF-NIK-p52/RelB axis is essential for B cell survival by upregulating Bcl-2 and Bcl-xl [65]. Additionally, TRAF3- or NIK-deficiency appeared to preclude T cell function by inhibiting the noncanonical NF-κB pathway [66, 67]. Intriguingly, the results from this study elucidated, for the first time, that tumour-derived RelB hampers T cell function by upregulating PD-L1. Thus, the implication of RelB in both immune cells and tumour cells has emerged as a major concern for tumour immunotherapy. Taken together, insight into RelB-mediated PD-L1 overexpression is anticipated to provide a promising approach for enhancing immune checkpoint blockade therapy through the administration of RelB.

## Conclusion

In summary, this study delineated that RelB participates in the regulation of PD-L1 in PCa cells. RelB and PD-L1 are highly expressed in advanced PCa and contribute to immune evasion. The silencing of RelB led to reduced PD-L1 expression and enhanced T cell immune response. Furthermore, RelB upregulates PD-L1 in response to cytokines, mainly through binding to a proximal NF-κB element located in the core promoter region of the *CD274* gene.

## Supplementary Information

**Additional file 1.**

**Additional file 2.**

**Additional file 3.**

**Additional file 4.**

**Additional file 5.**

**Additional file 6.**

**Additional file 7.**

**Additional file 8.**

**Additional file 9.**

## Data Availability

All data relevant to this study are included in the article and accessible online supplemental information. Data are available in a public, open access repository. Materials included in this study will be available for reasonable request to the corresponding author, Prof. Yong Xu.

## Abbreviations

PCa: Prostate cancer
mCRPC: Metastatic castration-resistant prostate cancer
TCGA: The Cancer Genome Atlas
PD-1: Programmed death receptor
PD-L1: A ligand of PD-1
CTLA-4: Cytotoxic T lymphocyte-associated antigen-4
NF-κB: Nuclear factor kappa light chain enhancer of activated B cells
IFN-γ: Human interferon-gamma
TNF-α: Tumour necrosis factor alpha
IL-2: Interleukin-2
shRNA: Small hairpin RNA
KO: Knockout
IHC: Immunohistochemistry
RNAseq: RNA sequencing
RT-qPCR: Reverse transcription-quantitative PCR
ChIP: Chromatin immunoprecipitation
EMSA: Electrophoretic mobility shift assay
RBC: Red blood cells
