# Supplementary material for: RelB upregulates PD-L1 and exacerbates prostate cancer immune evasion
Source: J Exp Clin Cancer Res. 2022 Feb 17;41:66. doi: 10.1186/s13046-022-02243-2 (PMC8851785; doi:10.1186/s13046-022-02243-2)
Supplement: Supplementary file 1 — Additional file 1. [file 13046_2022_2243_MOESM1_ESM.pdf]

Additional file 1

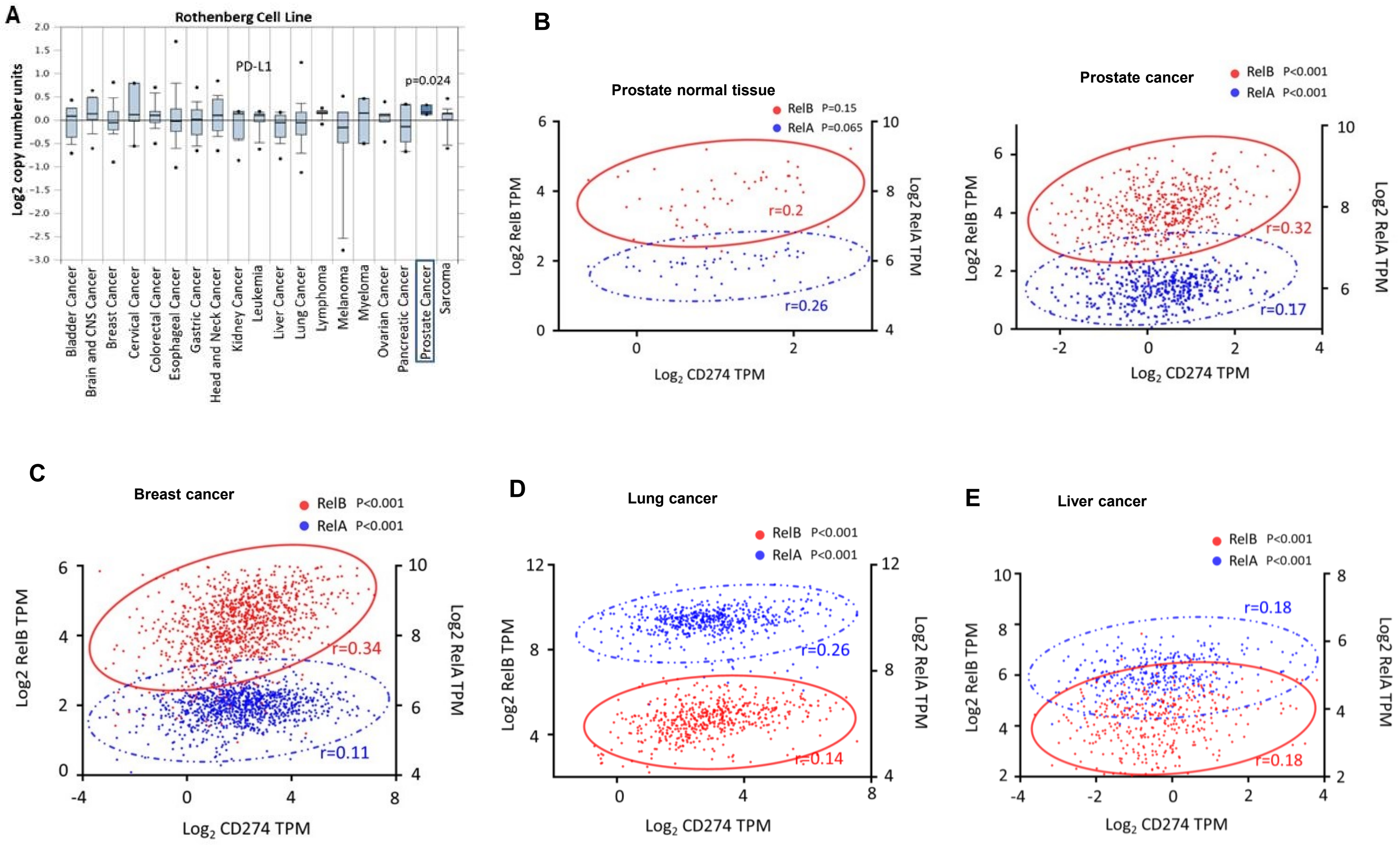

Fig. S1. The correlation of RelB and PD-L1 in Cancers. The association between RelB and PD-L1 in cancers was analysed using OncomineTM and TCGA public databases. **a** Per searching the PD-L1 expression profiles in various types of human cancer, PD-L1 is frequently elevated in tumour tissues compared to the corresponding peritumoral tissues. *($p < 0.05$) indicates the significance and the data in PCa is indicated in a box. **b** The correlation between RelA/RelB and PD-L1 in PCa vs. normal prostate tissues. **c** The correlation between RelA/RelB and PD-L1 in breast cancer. **d-e** The correlation between RelA/RelB and PD-L1 in lung and liver cancers.
