# Supplementary material for: RelB upregulates PD-L1 and exacerbates prostate cancer immune evasion
Source: J Exp Clin Cancer Res. 2022 Feb 17;41:66. doi: 10.1186/s13046-022-02243-2 (PMC8851785; doi:10.1186/s13046-022-02243-2)
Supplement: Supplementary file 2 — Additional file 2. [file 13046_2022_2243_MOESM2_ESM.pdf]

Additional file 2

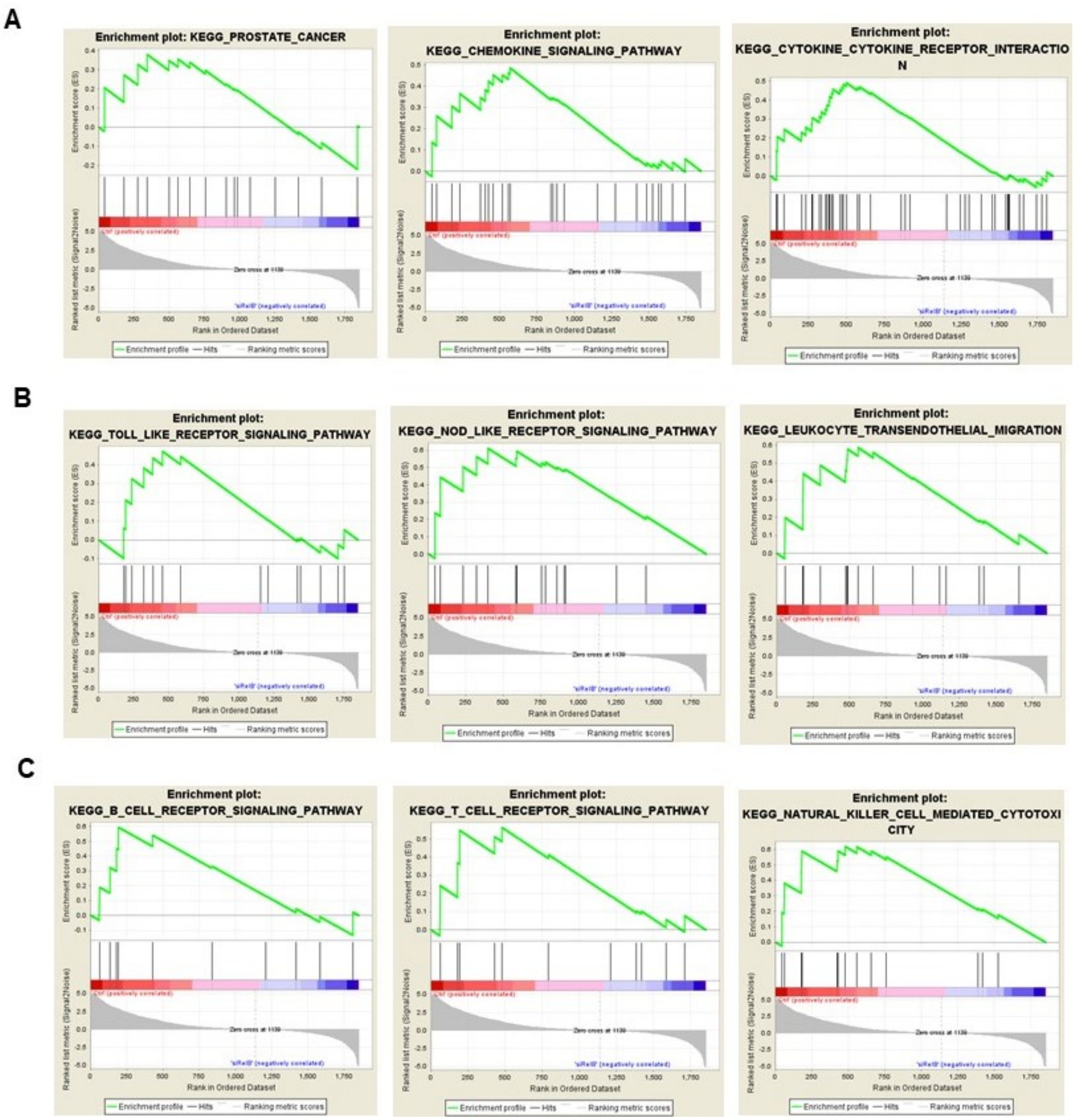

Fig. S2. Comparison of mRNA expression profiles in RelB-silenced PC-3 cells vs. control cells. Total RNA was extracted from shRelB and shCtrl PC-3 cells. The mRNA expression profiles were examined by RNA-Seq. **a** KEGG pathway enrichment analysis was performed to assess the association of altered mRNA profiles with cytokine/chemokine-activated signalling pathways. **b-c** The altered mRNA profiles are also related to oncogenic receptor-mediated signalling pathways and the immune response.
