# Supplementary material for: RelB upregulates PD-L1 and exacerbates prostate cancer immune evasion
Source: J Exp Clin Cancer Res. 2022 Feb 17;41:66. doi: 10.1186/s13046-022-02243-2 (PMC8851785; doi:10.1186/s13046-022-02243-2)
Supplement: Supplementary file 3 — Additional file 3. [file 13046_2022_2243_MOESM3_ESM.pdf]

Additional file 3

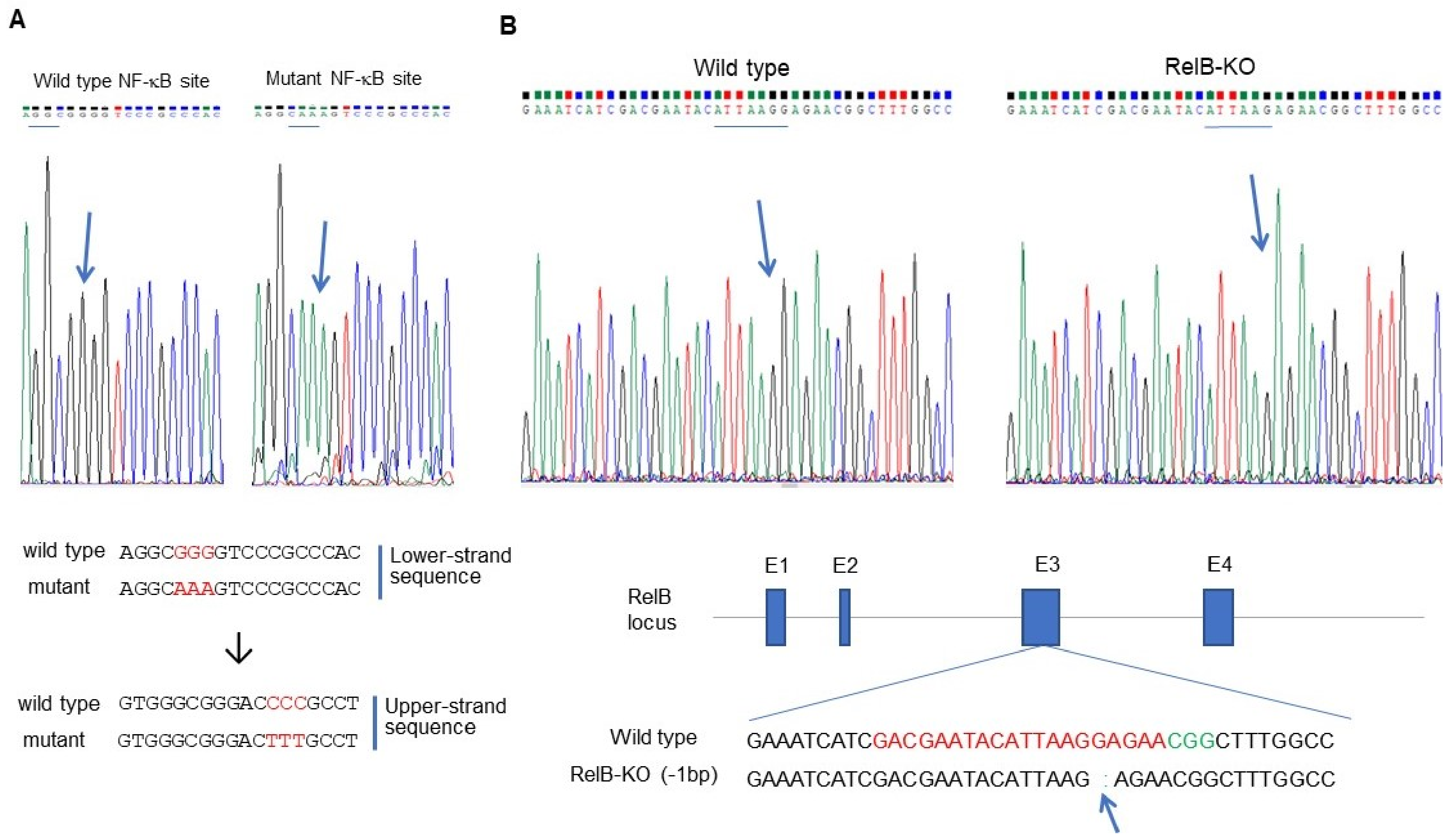

Fig. S3. Confirmation of plasmid constructs by DNA sequencing. a The proximal NF-kB binding site identified in the human *CD247* gene was mutated in the *luciferase* reporter plasmid. DNA sequencing was performed using a reverse primer, and the relative upper-strand sequence was converted to the lower panel. Arrows indicate the mutant site in the up panel. **b** RelB was knocked out in RM-1 cells using a CRISPR/Cas9-mediated gene edition system. Arrows indicate that the "G" was deleted in the RelB-KO construct, the sgRNA binding site is shown in green, and the target sequence is shown in red.
