# Supplementary material for: RelB upregulates PD-L1 and exacerbates prostate cancer immune evasion
Source: J Exp Clin Cancer Res. 2022 Feb 17;41:66. doi: 10.1186/s13046-022-02243-2 (PMC8851785; doi:10.1186/s13046-022-02243-2)
Supplement: Supplementary file 4 — Additional file 4. [file 13046_2022_2243_MOESM4_ESM.pdf]

Additional file 4

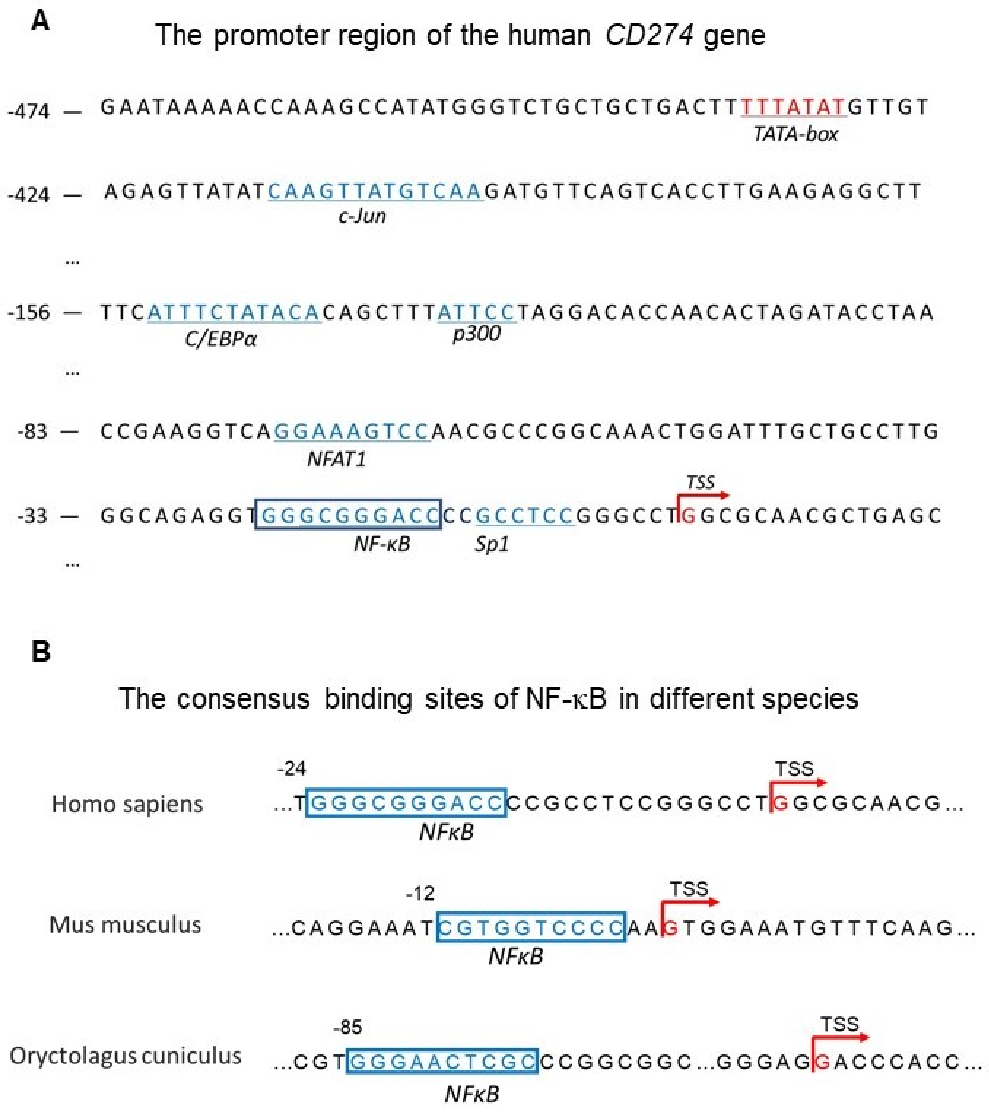

Fig. S4. Identification of the proximal NF-κB element in the *CD274* genes. **a** The sequence of the promoter region of the human *CD274* gene, **b** The conserved proximal NF-κB element in the *CD274* genes exists in different mammalian species. Transcription factors binding sites are boxed and the transcriptional start site (TSS) is indicated by red arrows.
