# Supplementary material for: RelB upregulates PD-L1 and exacerbates prostate cancer immune evasion
Source: J Exp Clin Cancer Res. 2022 Feb 17;41:66. doi: 10.1186/s13046-022-02243-2 (PMC8851785; doi:10.1186/s13046-022-02243-2)
Supplement: Supplementary file 5 — Additional file 5. [file 13046_2022_2243_MOESM5_ESM.pdf]

Additional file 5

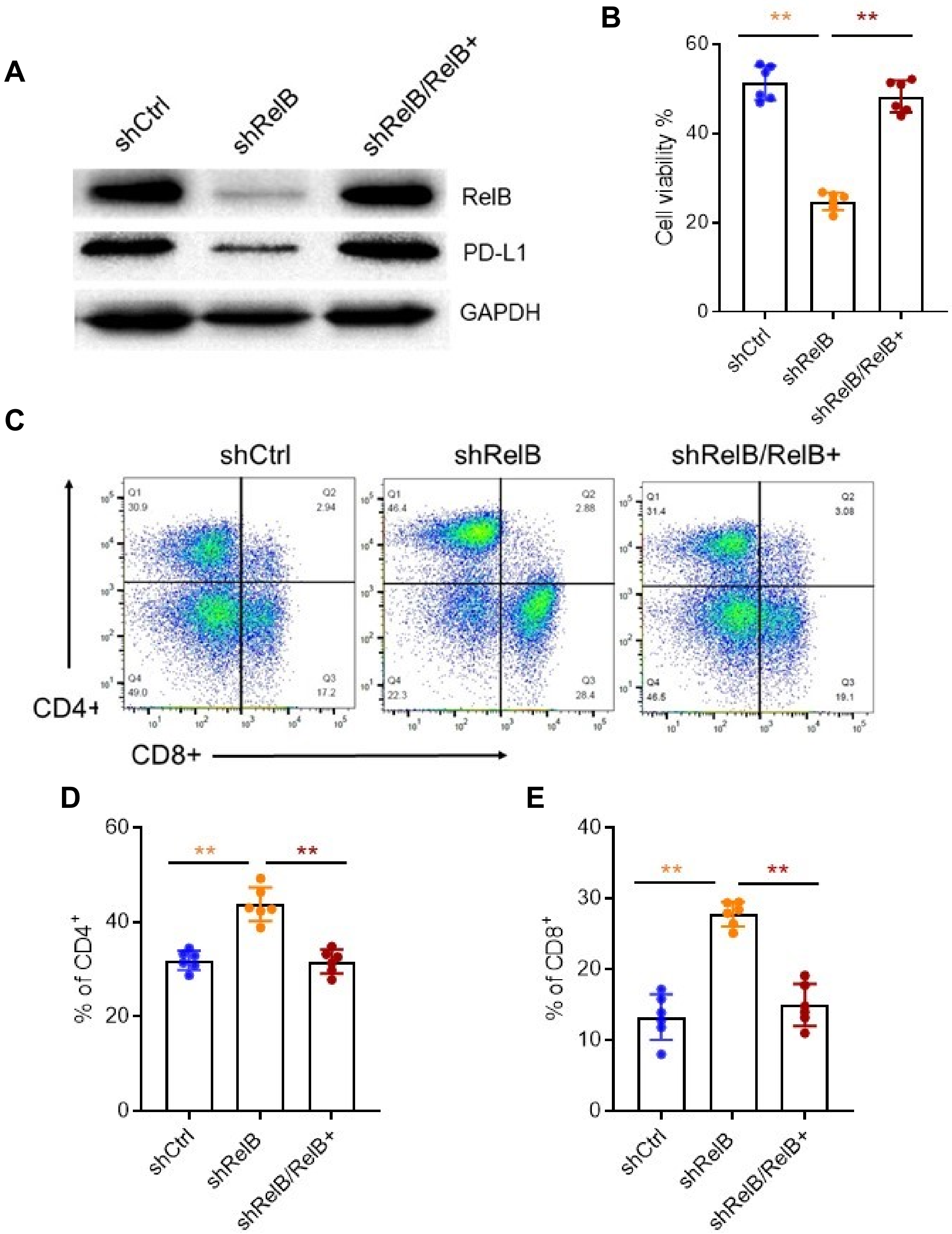

Fig. S5. Reversible immune evasion of PCa cells by restoring RelB. a A RelB cDNA expression construct was transiently transfected into the lentivirus-mediated RelB-silenced PC-3 cells. The cellular levels of RelB and PD-L1 were quantified by western blots, **b** PC-3 cells were cocultured with T cells isolated from blood samples donated from healthy donors. The survival fraction of the cancer cells was assessed using a CCK-8 assay. **c-e** In addition, after coculture, the percentages of CD4+ and CD8+ T cells were analysed by flow cytometry. **($p < 0.01$) shows significance between the two groups as indicated.
