# Supplementary material for: RelB upregulates PD-L1 and exacerbates prostate cancer immune evasion
Source: J Exp Clin Cancer Res. 2022 Feb 17;41:66. doi: 10.1186/s13046-022-02243-2 (PMC8851785; doi:10.1186/s13046-022-02243-2)
Supplement: Supplementary file 6 — Additional file 6. [file 13046_2022_2243_MOESM6_ESM.pdf]

Additional file 6

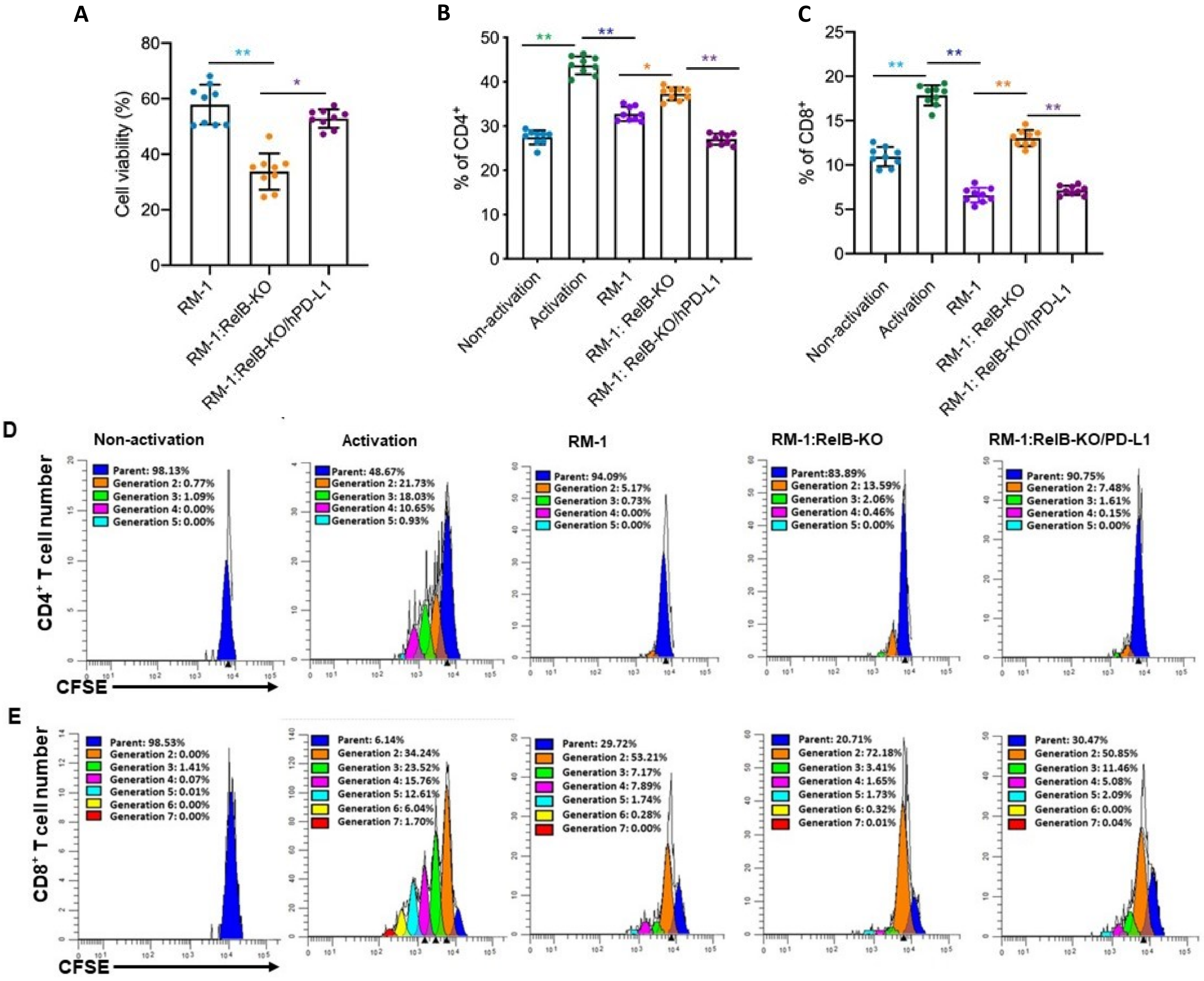

Fig. S6. Enhancement of $CD4^+$ and $CD8^+$ cells by knockout of RelB in RM-1 cells. a Activated mouse T cells were cocultured with RM-1 cell lines in which RelB and PD-L1 were manipulated. T-cell induced cytotoxicity in RM-1 cell lines was determined by MTT assay. **b-c** The effects of RM-1 cell lines on numbers of $CD4^+$ and $CD8^+$ cells were quantified by flow cytometry. **d-e** The proliferation of $CD4^+$ and $CD8^+$ cells was further analysed by flow cytometry. *($p < 0.05$) and **($p < 0.01$) show significance between the two groups as indicated.
