# Supplementary material for: RelB upregulates PD-L1 and exacerbates prostate cancer immune evasion
Source: J Exp Clin Cancer Res. 2022 Feb 17;41:66. doi: 10.1186/s13046-022-02243-2 (PMC8851785; doi:10.1186/s13046-022-02243-2)
Supplement: Supplementary file 7 — Additional file 7. [file 13046_2022_2243_MOESM7_ESM.pdf]

Additional file 7

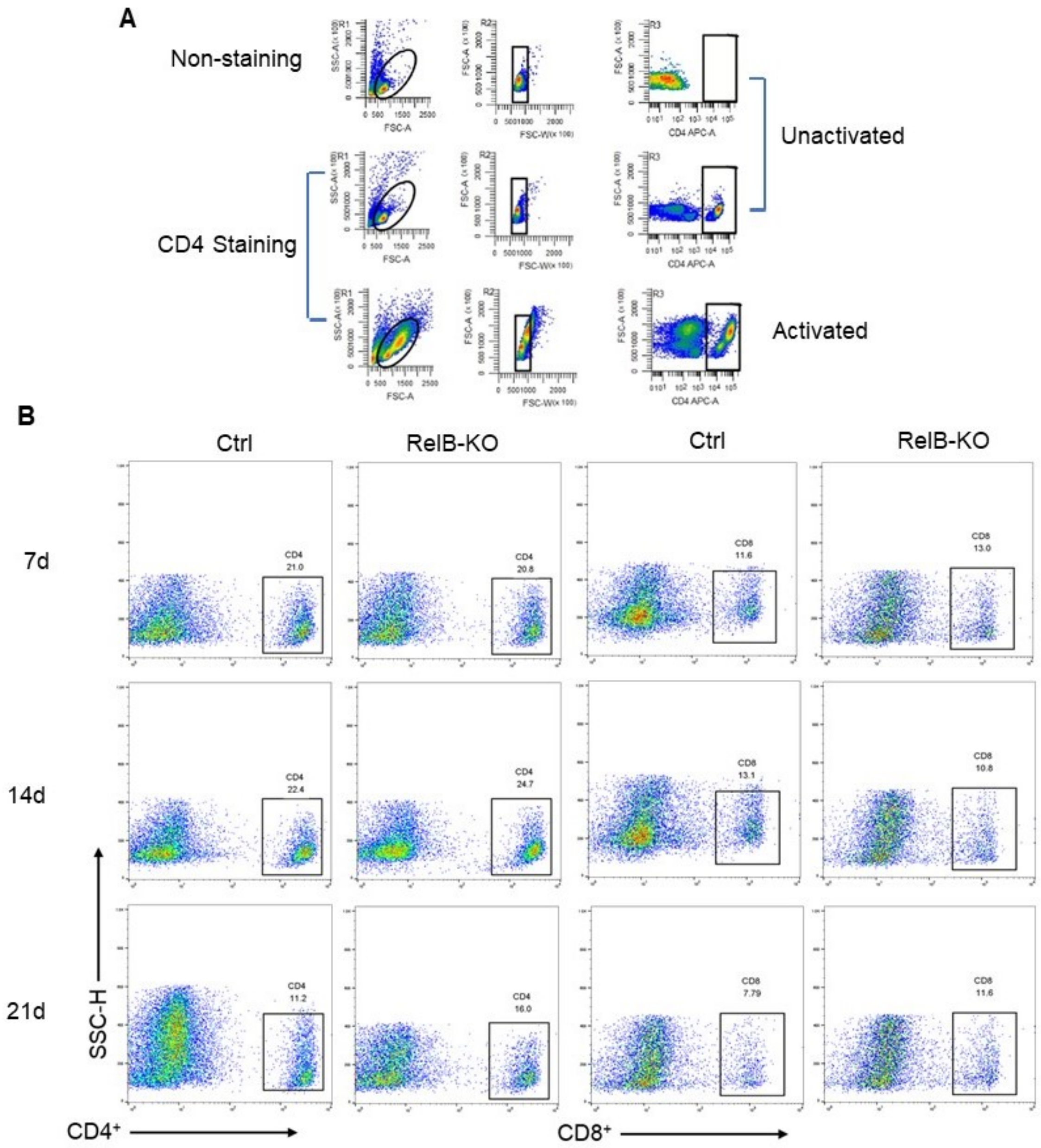

Fig. S7. Activation of mouse CD4$^+$ and CD8$^+$ cells by injection of RM-1 cells. a T cells were isolated from mouse blood samples and activated by CD3 and CD28 stimulation. The activated T cells were qualified by CD4 staining. **b** After RM-1 cell injection, blood samples were drawn from mice on different days as indicated. The fractions of CD4$^+$ and CD8$^+$ cells were examined by flow cytometry with relative antibodies.
