# Supplementary material for: RelB upregulates PD-L1 and exacerbates prostate cancer immune evasion
Source: J Exp Clin Cancer Res. 2022 Feb 17;41:66. doi: 10.1186/s13046-022-02243-2 (PMC8851785; doi:10.1186/s13046-022-02243-2)
Supplement: Supplementary file 8 — Additional file 8. [file 13046_2022_2243_MOESM8_ESM.pdf]

Additional file 8

Table S1. Sequences of RT-qPCR/ChIP primers and EMSA probes

RT-qPCR primer		Sequence
CD274		Forward: 5'- GGAGCCATCTTATTATGCCTT-3'<br>Reverse: 5'- GTTTGTATCTTGGATGCCACA-3'
GAPDH		Forward: 5'- TCTGACTTCAACAGCGACACC-3'<br>Reverse: 5'- CTGTTGCTGTAGCCAAATTCGTT-3'
**ChIP primer**	**Location**	**Sequence**
E1.	-1289	Forward: 5'-GCAGTACCTGTAAACTGTATTGCC-3'<br>Reverse: 5'-GTTCTCTTTGGCCCCAATAA-3'
E2.	-364	Forward: 5'-ATATGGGTCTGCTGCTGACTTT-3'<br>Reverse: 5'-ACAACAAGCCAACATCTGAACG-3'
E3.	-21	Forward: 5'-ATTTCACCGAAGGTCAGGAA-3'<br>Reverse: 5'-AGCGAGCTAGCCAGAGATACTG-3'
**EMSA probe**		**Sequence**
Wild type		Forward: 5’-AGAGGTGGGCGGACCCCGCCTC-3’<br>Reverse: 5’-GAGGCGGGGTCCCGCCCACCTCT-3’
Mutant		Forward: 5’-AGAGGTGTGCATCTGCATGCCTC-3’<br>Reverse: 5’-GAGGCATGCAGATGCACACCTCT-3’
