# Supplementary material for: RelB upregulates PD-L1 and exacerbates prostate cancer immune evasion
Source: J Exp Clin Cancer Res. 2022 Feb 17;41:66. doi: 10.1186/s13046-022-02243-2 (PMC8851785; doi:10.1186/s13046-022-02243-2)
Supplement: Supplementary file 9 — Additional file 9. [file 13046_2022_2243_MOESM9_ESM.pdf]

Additional file 9

Table S2. mRNA expression profiles in PC-3/shRelB cell vs. PC-3/shCtrl cell

Gene name	Ctrl (FPKM)	RelB-KO (FPKM)	log_2(RelB-KO/Ctrl)	up-or-down	P value
CXCL8	167.11±8.17	72.80±8.17	-1.20	down	5.00E-05
PTPN6	3.48±0.38	1.58±0.47	-1.14	down	5.00E-05
HLA-B	30.86±3.63	6.46±0.22	-2.26	down	5.00E-05
SERPINE1	3.75±0.36	0.40±0.21	-3.23	down	5.00E-05
CD274	0.67±0.04	0.12±0.04	-2.46	down	5.00E-05
EBI3	1.62±0.32	0.57±0.24	-1.50	down	5.00E-05
CCL2	10.35±0.94	0.00±0.00	#	down	5.00E-05
SHH	3.76±0.42	0.07±0.03	-5.79	down	5.00E-05
CAV1	131.27±1.75	35.20±0.57	-1.90	down	5.00E-05
PLA2G7	1.63±0.19	0.72±0.04	-1.19	down	5.00E-05
MMP14	21.12±1.20	9.99± 0.25	-1.08	down	5.00E-05
STAT6	10.67±0.63	0.71±0.12	-3.90	down	5.00E-05
INHBA	11.71±0.76	2.01±0.17	-2.54	down	5.00E-05
AXL	6.06±0.06	1.98±0.17	-1.62	down	5.00E-05
GPR68	0.94±0.12	0.14±0.06	-2.78	down	5.00E-05
PIK3AP1	0.66±0.10	0.06±0.01	-3.41	down	5.00E-05
PSMB9	1.94±0.51	0.69±0.31	-1.48	down	5.00E-05
HOXA5	0.47±0.04	0.11±0.00	-2.04	down	0.00465
C3	32.55±0.56	14.07±0.27	-1.21	down	5.00E-05
Fas	3.93±0.47	2.99±0.32	-0.40	down	0.06405
VNN1	12.35±0.41	40.31±2.58	1.71	up	5.00E-05
MYB	6.09±0.31	11.96±0.45	0.97	up	5.00E-05
CR2	0.88±0.13	2.09±0.16	1.24	up	5.00E-05
CD86	0.84±0.18	1.88±0.37	1.16	up	5.00E-05
TNFRSF18	1.25±0.44	3.82±0.13	1.62	up	5.00E-05
FCGR2A	3.71±0.26	7.46±0.40	1.01	up	5.00E-05
